# Pu isotopes in soils collected downwind from Lop Nor: regional fallout vs. global fallout

**DOI:** 10.1038/srep12262

**Published:** 2015-07-17

**Authors:** Wenting Bu, Youyi Ni, Qiuju Guo, Jian Zheng, Shigeo Uchida

**Affiliations:** 1 State Key Laboratory of Nuclear Physics and Technology, School of Physics, Peking University, Beijing 100871, China; 2 Research Center for Radiation Protection, National Institute of Radiological Sciences, Anagawa 4-9-1, Inage, Chiba 263-8555, Japan

## Abstract

For the first time, soil core samples from the Jiuquan region have been analyzed for Pu isotopes for radioactive source identification and radiological assessment. The Jiuquan region is in downwind from the Lop Nor Chinese nuclear test (CNT) site. The high Pu inventories (13 to 546 Bq/m^2^) in most of the sampling locations revealed that this region was heterogeneously contaminated by the regional fallout Pu from the CNTs. The contributions of the CNTs to the total Pu in soils were estimated to be more than 40% in most cases. The ^240^Pu/^239^Pu atom ratios in the soils ranged from 0.059 to 0.186 with an inventory-weighted average of 0.158, slightly lower than that of global fallout. This atom ratio could be considered as a mixed fingerprint of Pu from the CNTs. In addition, Pu in soils of Jiuquan region had a faster downward migration rate compared with other investigated places in China.

A total of 543 atmospheric nuclear tests took place worldwide from 1945 to 1980. As a result, large amounts of Pu isotopes (^239^Pu, ^240^Pu, ^241^Pu etc.) were released into the environment. The contamination of Pu isotopes could be local, regional (tropospheric) and global (stratospheric) depending on the power of the explosion, the explosion height and the meteorological conditions. Normally, most of the radioactive debris from high-yield explosions was injected into the lower or upper stratosphere, while that of the low-yield explosions stayed almost completely in the troposphere and deposited downwind from the nuclear test sites^1^.

Based on the time of the nuclear test programs, several distinct periods of atmospheric nuclear tests can be identified. The first period (1952–1958) was dominated by high-yield thermonuclear tests conducted by the US. After that, a series of large scale atmospheric nuclear tests was carried out by the former Soviet Union from 1961 to 1962. Since the Test Ban Treaty in 1962, smaller scale atmospheric nuclear tests were done by France and China. From 1964 to 1980, 22 atmospheric tests took place in the Lop Nor Chinese nuclear test (CNT) site^2^. Investigating Pu isotopes in the downwind areas from nuclear test sites is important with regard to radioactive source identification and radiological assessment. The distributions of Pu isotopes in the areas around the US’s Nevada nuclear test site and the former Soviet Union’s Semipalatinsk nuclear test site have been extensively studied^3^,^4^,^5^. Information about the contamination situation of Pu isotopes downwind from the CNT site is very limited^6^,^7^, however. Radioactive clouds containing hundreds of radionuclides formed after the tests and moved to the east passing over the Jiuquan region in Gansu Province, the closest downwind area with human habilitations to the CNT site^8^. Thus, additional radioactive contamination rather than just global fallout occurred in this region as regional fallout.

In order to assess the cumulative effective dose from the CNTs to the population, a large scale investigation was carried out for the analysis of radionuclides in soil samples collected in the Jiuquan region 20 years ago. The averaged total ^137^Cs inventory in this region was estimated to be 2.99 kBq/m^2^ and *ca.* 70% of that originated from the CNTs^8^. However, due to the limitation of the analytical method available at the time, Pu isotopes were not included in the investigation. The first study about Pu contamination in the soils around the CNT site was conducted by Zhang *et al.*^9^ for samples obtained in Xinjiang Province; alpha spectrometry was used for this work. The ^239+240^Pu activities in the surface soils in the upwind area from the CNT site ranged from 0.36 to 0.68 mBq/g and the inventories of Pu were comparable with that of global fallout. At the only sampling location in the downwind area, however, a high ^239+240^Pu inventory of 321.7 Bq/m^2^ was observed. As the isotopic compositions of Pu isotopes were not determined, the contribution of Pu from the CNTs could not be well identified.

In recent years, the applications of mass spectrometry had made it possible to determine the ^240^Pu/^239^Pu atom ratio as well as the ^239+240^Pu activity in various environmental samples^10^,^11^. The ^240^Pu/^239^Pu atom ratio that originates from different atmospheric nuclear tests varies depending on the yield of the test and the design of the bomb. For example, the ^240^Pu/^239^Pu atom ratios from the Semipalatinsk test site and the Nevada test site were found to be 0.025–0.072 and 0.054–0.063, respectively, while those of the Pacific Proving Grounds in the North Pacific Ocean were exceeded 0.30^5^,^12^,^13^,^14^. Therefore, the ^240^Pu/^239^Pu atom ratio is regarded as a useful indicator for Pu source identification. Compared with other nuclear test sites, the isotopic composition of Pu isotopes from the CNT site has not been well addressed.

Dong *et al.*^15^ determined Pu isotopes in soils collected in Xinjiang Province; the collection locations were in upwind areas 200–400 km from the CNT site. They found that the ^240^Pu/^239^Pu atom ratios were similar to the global fallout value (0.18). In Gansu Province, which is in the downwind direction from the CNT site, Jin *et al.*^16^ observed a low ^240^Pu/^239^Pu atom ratio of 0.16 in a soil sample. Zheng *et al.*^17^, however, studied the Pu distribution in a soil core sample collected from Lanzhou (*ca.* 1200 km from the CNT site) in Gansu Province and the determined ^240^Pu/^239^Pu atom ratio was 0.188 ± 0.009, revealing that global fallout was the main source for Pu contamination in their sampling place. Two other studies focused on the characterization of Pu in sediments from lakes in Gansu and Xinjiang Provinces and low ^240^Pu/^239^Pu atom ratios down to 0.08 were observed^18^,^19^. These studies suggested that the freshwater lakes around the CNT site received Pu deposition from the CNTs and the ^240^Pu/^239^Pu atom ratio from the CNTs could be lower than that of global fallout. However, a more comprehensive investigation with a wider sampling range is needed to better understand the contamination of Pu in the downwind area from the CNT site and the isotopic composition of Pu from the tests should be obtained.

In this study, ^239+240^Pu activities and ^240^Pu/^239^Pu atom ratios were analyzed in eight soil core samples collected in the Jiuquan region of Gansu Province (Fig. 1) downwind from the CNT site^20^. In three of the eight soil cores, the activities of ^137^Cs were also measured. The purposes of this work were to: (1) obtain basic information about the Pu distribution in the downwind area from the CNT site for radiological assessment; (2) provide the typical ^240^Pu/^239^Pu atom ratio value of the CNTs for Pu source identification; and (3) resolve the question of whether the fallout Pu is regional or global in this region from which the influence of the nuclear weapon tests on Pu contamination can be understood.

## Results

The obtained ^239+240^Pu and ^137^Cs activities, ^240^Pu/^239^Pu atom ratios and ^137^Cs/^239+240^Pu activity ratios in the soil samples are summarized in Table S1 in supporting information. Vertical distributions of ^239+240^Pu activities and ^240^Pu/^239^Pu atom ratios are illustrated in Fig. 2. The ^239+240^Pu activities in the soil samples showed a large variation, ranging from 0.026 to 2.697 mBq/g. For the three soil cores YM1, GZ2 and GZ3 that ^137^Cs activities were measured in, the ^137^Cs activities ranged from 1.3 to 21.2 mBq/g. In the soil cores GZ1, GZ2 and DH1, the highest ^239+240^Pu activities were observed in the surface soil and the ^239+240^Pu activities showed a decreasing trend with increasing soil depth. Subsurface maximums of ^239+240^Pu activity were found in the soil cores GZ3, DH2-1 and YM1. These two different vertical distributions of ^239+240^Pu activity have been reported for undisturbed soils in other regions of China and in South Korea as well^7^,^21^,^22^,^23^. In the other two soil cores DH2-2 and YM2, however, the ^239+240^Pu activities increased from the surface layer to the depth of 30 cm. The ^239+240^Pu inventories, calculated by integrating the Pu content in each layer (0–30 cm) in the soil cores were as follows: YM1, 190 ± 8 Bq/m^2^; YM2, 56 ± 5 Bq/m^2^; GZ1, 439 ± 12 Bq/m^2^; GZ2, 13 ± 1 Bq/m^2^; GZ3, 109 ± 7 Bq/m^2^; DH1, 122 ± 8 Bq/m^2^; DH2-1, 485 ± 12 Bq/m^2^; DH2-2, 546 ± 27 Bq/m^2^ (Fig. 3). It should be noted that these values may be underestimating the total Pu inventories of the sampling locations as Pu at some places has migrated to greater depths than 30 cm. Most of the Pu inventories were remarkably higher than the global fallout inventory (58 Bq/m^2^) within the 40°–50°N latitude band^2^, suggesting these locations received regional fallout Pu from the CNTs. Although the soil core samples were all collected in the Jiuquan region of Gansu Province, the ^239+240^Pu inventories varied significantly among the sampling locations. This large variation could be due to the heterogeneous deposition of Pu from the CNTs and the redistribution of soil at the sampling locations.

The ^240^Pu/^239^Pu atom ratios in all the soil core samples varied from 0.059 to 0.186 with an inventory-weighted average of 0.158 ± 0.011 (1σ) (Fig. 4). More specifically, the mean inventory-weighted ^240^Pu/^239^Pu atom ratios were: YM1, 0.132 ± 0.010; YM2, 0.168 ± 0.020; GZ1, 0.159 ± 0.008; GZ2, 0.163 ± 0.010; GZ3, 0.167 ± 0.015; DH1, 0.159 ± 0.007; DH2-1, 0.158 ± 0.007; and DH2-2, 0.164 ± 0.016. All these values were slightly lower than that (0.18) of global fallout^24^. Two extremely low ^240^Pu/^239^Pu atom ratios of 0.059 and 0.085 were observed for YM1 (20–25 cm) and YM2 (6–8 cm) locations, respectively. Similar results were also reported in lake sediments collected in northwest China and the CNTs were suggested to contribute to the Pu contamination in these lakes^18^,^19^. The present results for the soil core samples collected downwind from the CNT site further revealed that the ^240^Pu/^239^Pu atom ratios derived from the regional fallout of the CNTs could be lower than the global fallout value. For the three soil cores in which ^137^Cs activity was measured, the ^137^Cs/^239+240^Pu activity ratios ranged from 14.2 to 39.7 with an average of 26 ± 6 (1σ) (^137^Cs decay corrected to 15 October 2011, the core sampling date). Although the ^137^Cs/^239+240^Pu activity ratios varied, the mean ^137^Cs/^239+240^Pu activity ratio was similar to that (26 ± 3, ^137^Cs decay corrected to 15 October 2011) of global fallout^25^.

## Discussion

At the core sampling locations of the present study, two possible sources for Pu isotopes existed: global fallout from the worldwide atmospheric nuclear tests and regional fallout from the CNTs. As Pu isotopes were not included in the early radiation survey program of the 1990s conducted by the Chinese government in the downwind area from the Lop Nor CNT site, the range and level of Pu contamination caused by the tests within China remains unknown. Due to improvement of the analytical method for Pu determination and increased concerns in the scientific community and among the general public about the environmental behavior of Pu, several studies characterizing Pu in the environment of China have been conducted in recent years^7^,^9^,^17^,^21^, ^22^, ^23^,^26^. The results of related studies about Pu distribution in soils from different areas of China are summarized in Table 1.

**Figure 1:**
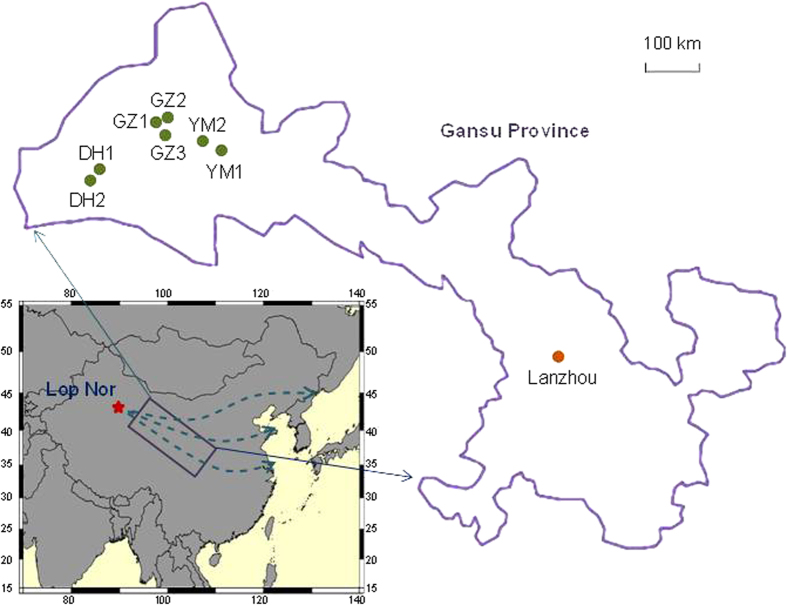
Map showing the soil core sampling locations. The Lanzhou location was studied in Ref. 17. The dashed lines represent the typical pathways of the radioactive clouds after the CNTs (cited from Ref. 20). This map was prepared with mapinfo professional 7.0 scp software.

For the sampling areas in this study, the highest ^239+240^Pu activity in the surface soil was found to be 1.99 mBq/g for the GZ1 core sample. It was considerably higher than even the highest value in the surface soils reported in other parts of China. The inventory of Pu in the soil core reflects the total amounts of Pu deposition in that area and could be estimated by summing the Pu activities in each layer. The ^239+240^Pu inventories in soils from the Jiuquan region varied significantly from 13 to 546 Bq/m^2^. All of them were significantly higher than that (58 Bq/m^2^) of the global fallout except for YM2 and GZ2. The fallout density of radionuclides from the atmosphere mainly depends on their concentrations in precipitation, the amount of precipitation in the time-interval of interest, the latitude, etc^27^. The Jiuquan region is in northwest China, and is dominated by a typical continental arid climate for which the annual precipitation is much lower than other parts of China. If global fallout was the only source for Pu deposition, the Pu inventory could be expected to be lower than the global fallout value due to the low wet deposition rate of Pu. For example, in Lanzhou city (Fig. 1), which is also located in Gansu Province but much further from the Lop Nor site, the ^239+240^Pu inventory in the soil was reported to be 32.4 Bq/m^2^ (0–23 cm)^17^. The abnormally high Pu inventories in the investigated area in this study strongly suggested that the CNTs introduced additional Pu deposition to that region. A similar high ^239+240^Pu inventory (321.7 Bq/m^2^) was also reported by Zhang *et al.*^9^ for samples from Xinjiang Province in the southwest direction from the CNT site; Xinjiang was in the radioactive cloud pathways of two small nuclear test explosions. It should be noted that the deposition of Pu from the CNTs in the downwind area seemed to be heterogeneous as the Pu inventories varied significantly among different sampling locations and a low ^239+240^Pu inventory down to 13 Bq/m^2^ was also observed for the GZ2 core sample.

The ^240^Pu/^239^Pu atom ratio varies depending on different sources (Table 2)^3^,^5^,^12^,^24^,^25^,^28^, ^29^, ^30^. Global fallout Pu has a ^240^Pu/^239^Pu atom ratio of 0.18, serving as an important baseline for the assessment of possible additional Pu inputs in the environment. For the CNTs, however, the ^240^Pu/^239^Pu atom ratio has not been well studied. A high ^240^Pu/^239^Pu atom ratio of 0.224 was found in the radioactive debris collected at the 10 km height in the atmosphere after the largest CNT (4 Mt) conducted on 17 November 1976^31^. High ^240^Pu/^239^Pu atom ratios above 0.2 were also observed in grass samples from the Rothamsted archive and the source of Pu was suggested to be the CNTs^32^. In China, however, several studies focusing on the characterization of Pu in soils and freshwater lake sediments around the CNT site obtained some low ^240^Pu/^239^Pu atom ratios. For example, Jin *et al.*^16^ reported a ^240^Pu/^239^Pu atom ratio of 0.16 in the soil of Gansu Province. More recently, Wu *et al.*^18^ and Liao *et al.*^19^ found ^240^Pu/^239^Pu atom ratios of 0.103 and 0.080 in deep sediments collected from Lake Sugan in the downwind direction from the CNT site and from Lake Boston in the upwind direction, respectively. These results suggested that the ^240^Pu/^239^Pu atom ratio from the CNTs could also be lower than the global fallout value.

The inventory-weighted ^240^Pu/^239^Pu atom ratios for the eight soil core samples collected downwind from the CNT site in the Jiuquan region were all lower than that of the global fallout. The individual ^240^Pu/^239^Pu atom ratios (0.059–0.186) of all the soil samples in the Jiuquan region together with that from soils in other regions of China are plotted in Fig. 4. Almost all the soil sample values were below the global fallout line. The lowest ^240^Pu/^239^Pu atom ratio was 0.059, comparable with that observed in the lake sediments in northwest China^18^,^19^. These results proved again that the soil samples were contaminated by regional fallout Pu from the CNTs. Usually, weapon-grade Pu is characterized by a ^240^Pu/^239^Pu atom ratio less than 0.07 as the nuclear fuel is left in the nuclear reactor for a short time to minimize the neutron activation of ^239^Pu during the production of Pu, while the ^240^Pu/^239^Pu atom ratios for the spent nuclear fuels were much higher, ranged from 0.2 to 0.8^33^, ^34^, ^35^. Because the Pu isotopic composition is changed only slightly during a low yield nuclear detonation, the ^240^Pu/^239^Pu atom ratio for the regional fallout could be similar to that of the nuclear weapon-grade Pu^24^. For example, the ^240^Pu/^239^Pu atom ratios in the soils around the Semipalatinsk and Nevada nuclear test sites were 0.025–0.072 and 0.054–0.063, respectively (Table 2)^5^,^12^. However, the situation for the CNT site is more complicated as the yields of the 22 atmospheric nuclear tests were over a wide range varying from 0.02 to 4 Mt. The 21st CNT (with the highest yield of 4 Mt) was characterized by high ^240^Pu/^239^Pu atom (0.224)^31^ and ^241^Pu/^239+240^Pu activity (11)^36^ ratios and it contributed mainly to the stratosphere fallout; thus no Pu from the 21st test was observed in the downwind area soil samples^2^. For all the soil samples analyzed in this study, the highest ^240^Pu/^239^Pu atom ratio was 0.186. Liao *et al.*^19^ studied the vertical distribution Pu isotopes in the sediments of Lake Boston in Xinjiang Province and found a ^240^Pu/^239^Pu atom ratio of 0.08 at a depth of 6 cm. Based on the chronology of the sediments, they suggested that this low atom ratio originated from the low yield tests conducted during the period from 1967 to 1973. Wu *et al.*^18^ also found a low ^240^Pu/^239^Pu atom ratio of 0.103 in sediments of Lake Sugan downwind from the CNT site. The two ^240^Pu/^239^Pu atom ratios below 0.1 found in the soils from Yumen City in the Jiuquan region (YM1 and YM2) were consistent with the results in the sediments of Lake Boston and Lake Sugan, revealing that the regional fallout Pu in the downwind area was present from the low yield tests CNTs. As discussed before, the ^240^Pu/^239^Pu atom ratio varied among the CNTs and it could be from 0.059 to 0.224. The regional fallout Pu isotopes from the CNTs and the global fallout Pu isotopes mixed in the soils of the Jiuquan region and showed a ^240^Pu/^239^Pu atom ratio of *ca.* 0.16, slightly lower than the global fallout value.

Normally, if Pu isotopes originate from two sources in a specific area, a two end-member mixing model can be used to estimate the contributions of these sources when the Pu isotopic compositions are well defined^37^,^38^. However, it is difficult to give an end-member ^240^Pu/^239^Pu atom ratio for the CNTs as was discussed before. In some cases, the activity ratio of ^137^Cs/^239+240^Pu has also been used for radionuclide source identifications and relative contribution calculations, because radiocesium and Pu isotopes were proven to have similar environmental behaviors in the terrestrial environment and were still fixed together even decades after their deposition in soils^4^,^21^,^25^. The ^137^Cs and ^239+240^Pu activities in the three soils cores YM1, GZ2 and GZ3 are plotted in Fig. 5. Their activities showed good consistency (R^2^=0.88) among the soil samples. The mean ^137^Cs/^239+240^Pu activity ratio in the investigated soils of the Jiuquan region was 26 ± 5 (decay corrected to 15 October 2011). Hodge *et al.*^25^ reported that the ^137^Cs/^239+240^Pu activity ratio for the global fallout was 26 ± 3 (decay corrected to 15 October 2011). By chance the ^137^Cs/^239+240^Pu activity ratio in the soils downwind from the CNT site was similar to that of the global fallout when taking into account the decay of ^137^Cs. Therefore, the contribution of the CNTs to Pu contamination in the Jiuquan region could not be calculated by either the ^240^Pu/^239^Pu atom ratio or the ^137^Cs/^239+240^Pu activity ratio. Here, a rough estimation was made using the excess Pu inventories in these soil cores without regard to the possible loss and accumulation of Pu in the soil due to resuspension. The global fallout Pu inventory of 58 Bq/m^2^ in the 40°–50°N north hemisphere band was regarded as the background level of Pu in Gansu Province^2^. Thus the contributed fractions from the CNTs could be estimated to be 69%, 87%, 47%, 52%, 88% and 89% for the YM1, GZ1, GZ3, DH1, DH2-1 and DH2-2 locations, respectively. Wu *et al.*^18^ also studied the contribution of the CNTs to Pu contamination in the sediments of Lake Sugan and Lake Shuangta in northwest China. Based on the sediment chronology information, contributions of Pu isotopes in these two lakes that originated from the CNTs were calculated to be 20–27%. In the Jiuquan region, Ren *et al.*^8^ investigated the ^137^Cs distribution in soils and lake sediments and they found that *ca.* 70% of ^137^Cs came from the CNTs. The present study results were higher than the estimate by Wu *et al.*^18^ and comparable with the results reported by Ren *et al.*^8^ for ^137^Cs.

Besides the contamination level and source identification of Pu in the environment, the migration behavior of Pu has also attracted considerable concerns for radiological assessment^22^,^39^. In South Korea, Lee *et al.*^23^ observed two types of Pu distribution patterns in soils: (1) the ^239+240^Pu activity decreased exponentially with increasing soil depth; and (2) the occurrence of a sub-surface maximum ^239+240^Pu activity followed by an exponential decline. These Pu distribution patterns have also been widely found in soils from central, northeast and southwest China^7^,^21^,^22^. In the Jiuquan region, Pu was similarly characterized in the soil cores of GZ1, GZ3, DH1 and DH2-1. For the other soil cores, however, the vertical distributions of Pu were more complicated.

In the Jiuquan region, the resuspension of surface soil is severe due to the dry weather, scarce vegetation and strong wind. Thus the concentration of Pu isotopes in the upper soil layers could be quite variable. For example, in the soil cores of YM2 and GZ2, Pu distributed almost evenly from surface to the investigated soil depth. ^239+240^Pu activities in these two soil cores were both relatively low (<0.3 mBq/g). The loss of Pu in the upper soil layers by resuspension could be the main reason for this phenomenon. Also, it should be noted that the GZ2 soil core was collected in a man-made forest, even though the sampling location was chosen to be in an open flat place, the artificial disturbance of soil during the planting process could be another potential reason for the uniform distribution of Pu and Cs in the GZ2 soil core. In the soil core of YM1, Pu distributed almost evenly from surface to 30 cm as well except at the depth of 20–25 cm where the highest ^239+240^Pu activity (1.184 ± 0.031 mBq/g) and the lowest ^240^Pu/^239^Pu atom ratio (0.085 ± 0.002) were found. The YM1 soil core was collected in a cultivated land and the redistribution of soil by human activities was expected. As we discussed before, Pu isotopes in the soils of the Jiuquan region was mixed by global fallout and the CNTs regional fallout. However, this mixing process was heterogeneous considering the different time of global fallout and the CNTs regional fallout. In the soil sample at the depth of 20–25 cm in the YM1 soil core, high portion of Pu isotopes from the CNTs regional fallout was observed, which leaded to the high ^239+240^Pu activity and low ^240^Pu/^239^Pu atom ratio. The low ^240^Pu/^239^Pu atom ratio (0.059 ± 0.005) at the depth 6–8 cm in the YM2 soil core could be caused by the same reason.

**Figure 2:**
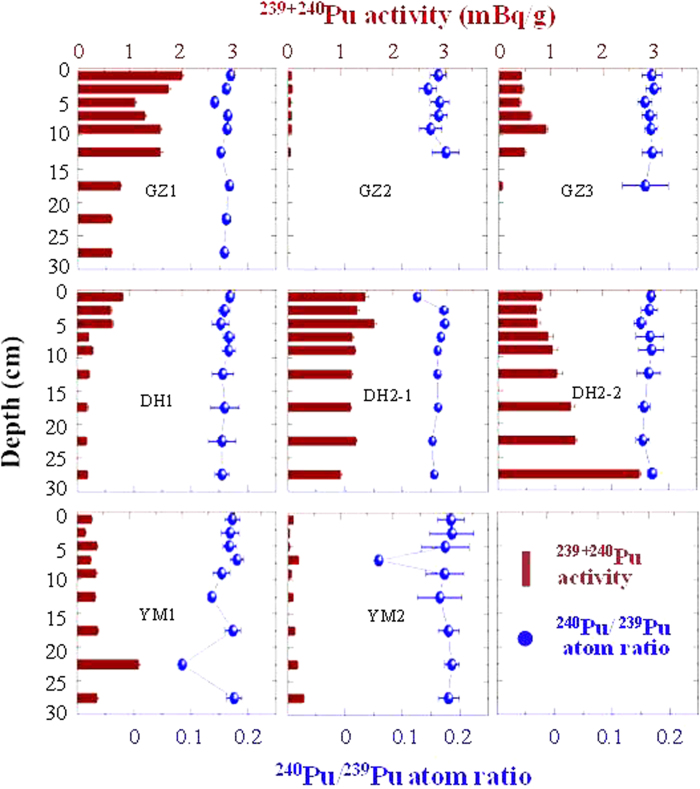
Vertical distributions of ^239+240^Pu activities and ^240^Pu/^239^Pu atom ratios in the soil cores.

**Figure 3:**
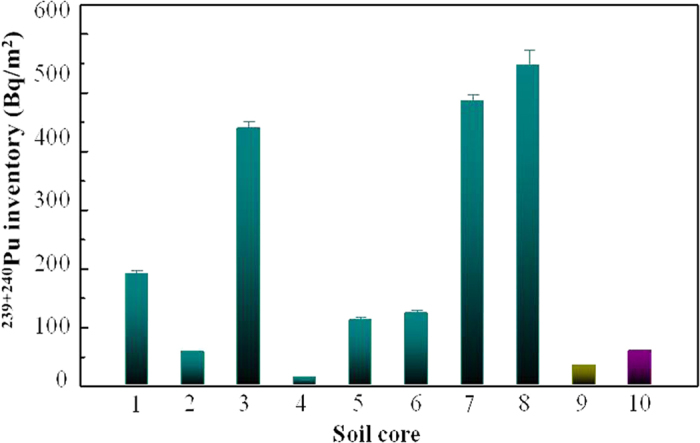
^239+240^Pu inventories in the Gansu soil cores. 1, YM1; 2, YM2; 3, GZ1; 4, GZ2; 5, GZ3; 6, DH1; 7, DH2-1; 8, DH2-2; 9, Lanzhou (cited from Ref. 17); 10, global fallout (cited from Ref. 2).

**Figure 4:**
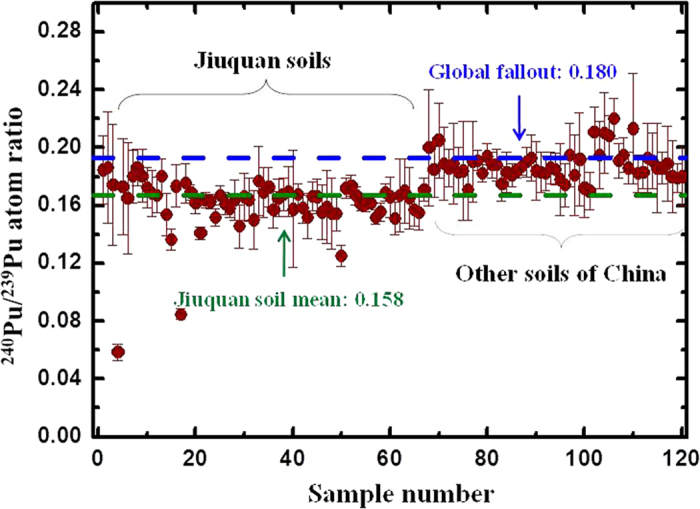
^240^Pu/^230^Pu atom ratios in Jiuquan soil samples and other soil samples from China. The two dashed lines represent the inventory-weighted average ^240^Pu/^230^Pu atom ratio of 0.158 and the global fallout value of 0.18. Data for the other soil samples include soils from Lanzhou in Northwest China (Ref. 17), Chongqing and Guizhou in Southwest China (Ref. 22), Hubei in Central China (Ref. 7) and Dalian in Northeast China (Ref. 21).

**Figure 5:**
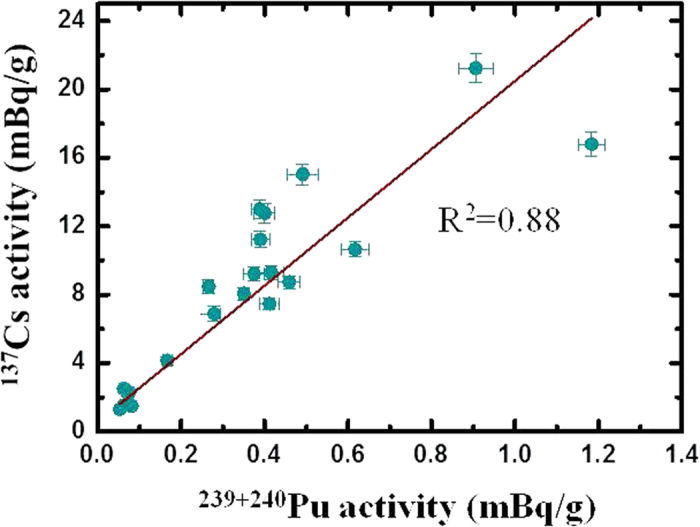
^239+240^Pu activity vs. ^137^Cs activity in the soil cores YM1, GZ2 and GZ3.

In a previous study, the migration of global fallout Pu in forest soils in southwest China was investigated and it was found that more than 70% of the Pu still remained in the top 10 cm layer of the soils even five decades after the deposition^22^. Pu in the soil cores of DH2-1 and DH2-2, however, showed a faster migration rate than that was observed in the forest soils. It can be seen in Fig. 2 that some of the Pu in these two locations has obviously migrated to depths greater than 30 cm. Especially for the soil core of DH2-2, Pu activities showed an increasing trend with increasing soil depth. The highest ^239+240^Pu activity in the DH2-2 soil core occurred in the soil at the 25–30 cm or deeper layer. Zheng *et al.*^17^ also observed a relatively fast downward migration of Pu in a soil core from Lanzhou in Gansu Province and the maximum of ^239+240^Pu was found to be at depths greater than 10 cm in 2007. The migration of Pu in soil is mainly controlled by the precipitation, the organic matter content and the mineral composition in the soil, the chemical properties of the soil solution, the micro-organism and fungi bioturbation, etc^40^,^41^,^42^. Considering the annual precipitation in the Jiuquan region is 37–154 mm, much lower than that (>1000 mm) in southwest China, the relatively low organic matter content (0.8–7.5%) could be one of the factors that weaken the Pu adsorption by soil and enhance the Pu downward migration in the place of DH2. However, further studies are needed to verify the specific migration mechanisms of Pu in soils of this region.

For the first time, this report describes the vertical distribution of ^239+240^Pu activities and ^240^Pu/^239^Pu atom ratios in soils from the Jiuquan region downwind from the CNT site. The high Pu inventories revealed that the Jiuquan region received Pu contamination from the CNTs and the Pu deposition was heterogeneous. Furthermore, in most cases, the contribution of Pu was more than 40%. The ^240^Pu/^239^Pu atom ratios in the soils downwind from the CNT site ranged from 0.059 to 0.186, with an inventory-weighted average of 0.158. Pu in the soils from the Jiuquan region showed a faster vertical migration rate compared with other places in China.

## Methods

### Soil sampling

Eight soil core samples, two (YM1, YM2) in Yumen City, three (GZ1, GZ2, GZ3) in Guazhou County, and three (DH1, DH2-1, DH2-2) in Dunhuang City, were collected in Jiuquan region, *ca.* 300–500 km east of Lop Nor, in the northwest of Gansu Province in July 2011 (Fig. 1). Jiuquan region is the nearest region with human habilitations downwind from the CNT site and it is dominated by a typical continental arid climate with an annual precipitation of 37–154 mm and temperature range of 3.9–9.3 °C^43^. The YM1 soil core was collected in a cultivated land with fine black soil covered by tinny grass and the YM2 soil core was collected in a small forest about 50 meters away from the provincial road. The GZ1 and GZ3 soil cores were collected in semidesert areas with brown soil covered by a small number of jujube trees. During the sampling of the GZ3 soil core, small stones were observed distributing in the top 30 cm layer. The GZ2 soil core was collected in a man-made forest with white poplar and this area has not been artificially disturbed for at least the last 20 years. In Dunhuang city, two places were chosen for soil sampling. The DH1 soil core was collected in an open area about 5 km from the Dunhuang airport with wet brown soil covered by rose willow. The second sampling site in Dunhuang city was located in a dense wood outside the Mogao Cave and the land was covered with thick fallen leaves. In this site, two soil cores were collected, separated by a distance of *ca.* 50 m. Each soil core was collected to a depth of 30 cm. The top 0–10 cm layer of soil was sliced at 2 cm intervals and 5 cm intervals for the 10–30 cm layer. For each soil core, small portions from different depths were taken out and mixed for the analysis of particle size distribution with a laser particle size analyzer (Mastersize 2000, Malvern Instruments, UK). The detailed information about the sample locations and soil particle size distributions is given in Table S2.

### Soil analysis for radiocesium and Pu isotopes

The soil samples were first dried at 105 °C for 24 h and then passed through a 20 mesh sieve to remove coarse stones. Then they were ashed in a muffle furnace at 450 °C for 5 h to decompose the organic matter. The organic matter content was calculated by the weight loss before and after the ashing procedure. The activity of ^137^Cs was measured in soil samples from the three locations YM1, GZ2 and GZ3 by a gamma spectrometer (Canberra BEGe, relative detection efficiency *ca.* 20%) before the analysis of Pu isotopes.

The chemical preparation procedures of soil samples for Pu analysis were revised from a previous work^44^. Briefly, a solid sample of about 1–3 g was weighed out and 0.57 pg ^242^Pu was added as a yield tracer. Acid digestion (with 20 mL conc. HNO_3_-1 M HF) was done on a hot plate at 160 °C for at least 4 h. After evaporating the filtered sample solution to dryness, 20 mL 8 M HNO_3_ was added to dissolve the sample residue. Then 0.28 g NaNO_2_ was added and the sample solution was heated at 40 °C for 30 min to convert Pu to the tetravalent state. After that, 0.3 g boric acid was added and the sample solution was heated at 80 °C for another 30 min to prevent the formation of Pu fluoride complex. A two-stage anion-exchange chromatography method using AG 1×8 and AG MP-1M resins was employed for the separation of Pu from the sample matrix and the further purification of Pu. The final sample solution was dissolved in 0.8 mL 4% HNO_3_, in preparation for Pu analysis. The overall chemical recovery was *ca.* 60%. The chemical procedure flow chart is shown in Fig. S1.

We used a sector field (SF)-ICP-MS (Element 2, Thermo Finnigan, Bremen, Germany) in a low resolution mode (m/△m = 300) for the analysis of Pu isotopes. A high efficiency sample introduction system (APEX-Q, Elemental Scientific Inc., Omaha, NE, USA) combined with a conical concentric nebulizer was used to improve the transportation efficiency of samples to the instrument. The isotopes of interest (^238^U, ^239^Pu, ^240^Pu, ^242^Pu) were analyzed in the peak hopping mode and the peak tops of the masses were measured at 10% of their respective peak width. The detailed operational setup and parameters of the sample introduction system and the SF-ICP-MS for Pu analysis have been given previously^45^. The sensitivity (^238^U) for this system was about 1.2×10^7^ cps ppb^−1^ and the detection limit of Pu was as low as 0.14 fg mL^−1^. A Pu isotope standard solution (NBS-947) with a known ^240^Pu/^239^Pu atom ratio of 0.242 was used for mass bias correction and two soil reference materials (IAEA-soil-6 and IAEA-375) were used for method validation.

## Additional Information

**How to cite this article**: Bu, W. *et al.* Pu isotopes in soils collected downwind from Lop Nor: regional fallout vs. global fallout. *Sci. Rep.* **5**, 12262; doi: 10.1038/srep12262 (2015).

## Supplementary Material

Supplementary Information

## Figures and Tables

**Table 1:** Vertical distributions of Pu isotopes in soils in different areas of China.

**Sample location**		**Surface ^239+240^Pu activity (mBq/g)**	**^240^Pu/^239^Pu atom range**	**^239+240^Pu inventory (Bq/m^2^)**	**Reference**
North China	Beijing	0.066–0.149		10.1–35.7	26
East China	Jinan	0.119–0.174		33.9 –36.4	26
Northeast China	Liaodong Bay	0.023–0.938	0.145–0.245	44.1–86.9	21
Central China	Hubei	0.358–0.380	0.135–0.220	44.9–54.6	7
Southwest China	Chongqing	0.171	0.170–0.206	19	22
	Guizhou	0.316–1.30	0.175–0.209	63–114	23
Northwest China	Xinjiang	0.363–0.927		76.6–321.7	9
	Lanzhou	0.023	0.171–0.205	32.4	17
	Jiuquan	0.076–1.988	0.059–0.186	13–546	This study

**Table 2:** Summary of ^240^Pu/^239^Pu atom ratio and ^137^Cs/^239+240^Pu activity ratio in soils contaminated by different sources.

**Sources**	**^240^Pu/^239^Pu atom ratio**	**^137^Cs/^239+240^Pu activity ratio**	**Reference**
Global fallout	0.180 ± 0.014	38 ± 4 (1 July 1994)	24,25
Semipalatinsk nuclear test site	0.025–0.072	11–16 (1 July 1998)	5
Nevada nuclear test site	0.054–0.063	4 (1 July 1998)	3,12
Chernobyl accident	0.4	106 ± 14 (I June 1997)	28
Fukushima accident	0.30–0.33	4×10^6^ - 2×10^7^ (11 March 2011)	29,30
CNT site	0.059–0.224	26 ± 6 (15 October 2011)	This study
