# Supplementary material for: Pu isotopes in soils collected downwind from Lop Nor: regional fallout vs. global fallout
Source: Sci Rep. 2015 Jul 17;5:12262. doi: 10.1038/srep12262 (PMC4505309; doi:10.1038/srep12262)
Supplement: Supplementary Information [file srep12262-s1.doc]

Supporting information

**Pu isotopes in soils collected downwind from Lop Nor: regional fallout vs. global fallout**

**Wenting Bu^1,2^, Youyi Ni^1^, Qiuju Guo^1*^, Jian Zheng^2*^, Shigeo Uchida^2^**

^1^State Key Laboratory of Nuclear Physics and Technology,

School of Physics, Peking University,

Beijing 100871, China

^2^Research Center for Radiation Protection,

National Institute of Radiological Sciences, Anagawa 4-9-1, Inage,

Chiba 263-8555, Japan

*Corresponding authors

Tel: 86-010-6275-5201 Fax: 86-010-6275-5403

Email: [qjguo@pku.edu.cn](mailto:qjguo@pku.edu.cn)

Tel: 81-043-206-4634 Fax: 81-043-255-0721

Email: [jzheng@nirs.go.jp](mailto:jzheng@nirs.go.jp)

Table S1. Analytical results for Pu and ^137^Cs isotopes in the soil core samples.

| **Sampling location** | **Depth**  **(cm)** | **Organic matter**  **(%)** | **^137^Cs activity**  **(mBq/g)**^a^ | **^239+240^Pu activity**  **(mBq/g)** | **^137^Cs/^239+240^Pu**  **activity ratio**^a^ | **^240^Pu/^239^Pu**  **atom ratio** | **^239+240^Pu inventory**  **(Bq/m^2^)** |
| --- | --- | --- | --- | --- | --- | --- | --- |
| YM1 | 0-2 | 3.5 | 6.9±0.4 | 0.280±0.016 | 24.6±2.1 | 0.172±0.012 | 190±8 |
|  | 2-4 | 2.5 | 4.2±0.2 | 0.166±0.011 | 24.9±2.1 | 0.168±0.019 |  |
|  | 4-6 | 3.2 | 13.0±0.6 | 0.389±0.019 | 33.4±2.2 | 0.167±0.013 |  |
|  | 6-8 | 4.0 | 8.5±0.4 | 0.266±0.013 | 31.8±2.2 | 0.180±0.013 |  |
|  | 8-10 | 2.6 | 9.2±0.4 | 0.375±0.025 | 24.6±2.0 | 0.154±0.009 |  |
|  | 10-15 | 2.8 | 8.1±0.4 | 0.351±0.012 | 23.0±1.3 | 0.136±0.007 |  |
|  | 15-20 | 3.4 | 12.8±0.6 | 0.400±0.025 | 31.9±2.4 | 0.173±0.014 |  |
|  | 20-25 | 4.2 | 16.8±0.7 | 1.184±0.031 | 14.2±0.7 | 0.085±0.002 |  |
|  | 25-30 | 3.5 | 11.2±0.5 | 0.390±0.022 | 28.8±2.0 | 0.175±0.013 |  |
| YM2 | 0-2 | 1.5 |  | 0.103±0.010 |  | 0.172±0.012 | 56±5 |
|  | 2-4 | 2.4 |  | 0.037±0.006 |  | 0.168±0.019 |  |
|  | 4-6 | 2.2 |  | 0.026±0.005 |  | 0.174±0.059 |  |
|  | 6-8 | 5.7 |  | 0.204±0.010 |  | 0.059±0.005 |  |
|  | 8-10 | 2.2 |  | 0.065±0.009 |  | 0.173±0.027 |  |
|  | 10-15 | 1.9 |  | 0.088±0.015 |  | 0.165±0.049 |  |
|  | 15-20 | 1.4 |  | 0.128±0.009 |  | 0.180±0.020 |  |
|  | 20-25 | 1.8 |  | 0.182±0.009 |  | 0.186±0.022 |  |
|  | 25-30 | 1.8 |  | 0.289±0.022 |  | 0.180±0.015 |  |
| GZ1 | 0-2 | 2.8 |  | 1.99±0.04 |  | 0.169±0.005 | 439±12 |
|  | 2-4 | 2.0 |  | 1.73±0.05 |  | 0.161±0.007 |  |
|  | 4-6 | 2.4 |  | 1.10±0.02 |  | 0.141±0.004 |  |
|  | 6-8 | 1.6 |  | 1.28±0.03 |  | 0.164±0.005 |  |
|  | 8-10 | 2.9 |  | 1.58±0.03 |  | 0.163±0.005 |  |
|  | 10-15 | 2.4 |  | 1.59±0.04 |  | 0.152±0.006 |  |
|  | 15-20 | 2.0 |  | 0.818±0.023 |  | 0.167±0.006 |  |
|  | 20-25 | 0.8 |  | 0.640±0.022 |  | 0.161±0.008 |  |
|  | 25-30 | 2.2 |  | 0.644±0.019 |  | 0.158±0.007 |  |
| GZ2 | 0-2 | 2.7 | 2.3±0.1 | 0.076±0.005 | 30.3±2.4 | 0.164±0.014 | 13±1 |
|  | 2-4 | 2.6 | 1.5±0.1 | 0.082±0.006 | 18.3±1.8 | 0.146±0.015 |  |
|  | 4-6 | 3.0 | 2.5±0.2 | 0.063±0.004 | 39.7±4.1 | 0.166±0.016 |  |
|  | 6-8 | 3.1 | 2.2±0.1 | 0.073±0.005 | 30.1±2.5 | 0.164±0.015 |  |
|  | 8-10 | 3.9 | 2.3±0.2 | 0.071±0.007 | 32.4±4.3 | 0.150±0.020 |  |
|  | 10-15 | 4.8 | 1.3±0.1 | 0.053±0.005 | 24.5±3.0 | 0.177±0.024 |  |
| GZ3 | 0-2 | 7.5 | 9.3±0.4 | 0.416±0.031 | 22.4±1.9 | 0.169±0.015 | 109±7 |
|  | 2-4 | 4.7 | 8.7±0.4 | 0.459±0.026 | 19.0±1.3 | 0.173±0.013 |  |
|  | 4-6 | 5.2 | 7.5±0.3 | 0.412±0.023 | 18.1±1.3 | 0.157±0.012 |  |
|  | 6-8 | 7.1 | 10.7±0.5 | 0.617±0.034 | 17.3±1.2 | 0.165±0.008 |  |
|  | 8-10 | 6.4 | 21.2±0.8 | 0.906±0.042 | 23.4±1.4 | 0.168±0.013 |  |
|  | 10-15 | 5.2 | 15.0±0.6 | 0.491±0.037 | 30.6±2.6 | 0.170±0.016 |  |
|  | 15-20 | 4.6 | 1.5±0.1 | 0.062±0.011 | 24.3±4.7 | 0.158±0.048 |  |
| DH1 | 0-2 | 7.0 |  | 0.858±0.027 |  | 0.168±0.008 | 122±8 |
|  | 2-4 | 5.0 |  | 0.651±0.030 |  | 0.158±0.011 |  |
|  | 4-6 | 6.7 |  | 0.670±0.045 |  | 0.152±0.010 |  |
|  | 6-8 | 3.9 |  | 0.228±0.009 |  | 0.166±0.011 |  |
|  | 8-10 | 3.7 |  | 0.295±0.019 |  | 0.166±0.011 |  |
|  | 10-15 | 3.1 |  | 0.225±0.019 |  | 0.155±0.016 |  |
|  | 15-20 | 3.2 |  | 0.202±0.022 |  | 0.159±0.012 |  |
|  | 20-25 | 2.3 |  | 0.177±0.019 |  | 0.154±0.023 |  |
|  | 25-30 | 2.9 |  | 0.198±0.011 |  | 0.154±0.013 |  |
| DH2-1 | 0-2 | 3.6 |  | 1.47±0.06 |  | 0.125±0.007 | 485±12 |
|  | 2-4 | 3.2 |  | 1.32±0.04 |  | 0.172±0.007 |  |
|  | 4-6 | 2.7 |  | 1.63±0.05 |  | 0.174±0.007 |  |
|  | 6-8 | 3.2 |  | 1.22±0.03 |  | 0.167±0.006 |  |
|  | 8-10 | 3.6 |  | 1.27±0.03 |  | 0.161±0.005 |  |
|  | 10-15 | 3.4 |  | 1.21±0.03 |  | 0.161±0.006 |  |
|  | 15-20 | 2.2 |  | 1.19±0.02 |  | 0.162±0.004 |  |
|  | 20-25 | 1.6 |  | 1.29±0.03 |  | 0.152±0.005 |  |
|  | 25-30 | 2.6 |  | 1.01±0.03 |  | 0.155±0.006 |  |
| DH2-2 | 0-2 | 5.3 |  | 0.833±0.022 |  | 0.169±0.006 | 546±27 |
|  | 2-4 | 7.1 |  | 0.736±0.073 |  | 0.166±0.014 |  |
|  | 4-6 | 6.7 |  | 0.744±0.071 |  | 0.151±0.011 |  |
|  | 6-8 | 5.1 |  | 0.956±0.101 |  | 0.167±0.025 |  |
|  | 8-10 | 4.9 |  | 1.04±0.11 |  | 0.170±0.022 |  |
|  | 10-15 | 4.5 |  | 1.12±0.10 |  | 0.165±0.021 |  |
|  | 15-20 | 3.7 |  | 1.39±0.08 |  | 0.157±0.011 |  |
|  | 20-25 | 3.1 |  | 1.47±0.04 |  | 0.155±0.012 |  |
|  | 25-30 | 2.2 |  | 2.70±0.04 |  | 0.171±0.008 |  |

^a^For ^137^Cs, decay was corrected to 15 October 2011, the soil sampling date.

Table S2. Locations of the soil core samples and soil particle size distributions.

| **Soil core** | **Location** | **Clay (%)** | **Silt (%)** | **Sand (%)** |
| --- | --- | --- | --- | --- |
| YM1 | 40º02'39''N 97º24'09''E | 13.7 | 82.0 | 4.3 |
| YM2 | 40º21'13''N 97º02'45''E | 7.0 | 71.6 | 21.4 |
| GZ1 | 40º32'43''N 95º48'50''E | 16.3 | 74.5 | 9.2 |
| GZ2 | 40º32'22''N 95º46'27''E | 15.9 | 73.4 | 10.7 |
| GZ3 | 40º24'33''N 95º44'57''E | 12.5 | 80.8 | 6.8 |
| DH1 | 40º09'48''N 94º53'24''E | 4.3 | 53.0 | 42.7 |
| DH2-1 | 40º03'44''N 94º47'33''E | 10.1 | 74.4 | 15.4 |
| DH2-2 | 40º03'44''N 94º47'33''E | 9.8 | 78.2 | 12.0 |

Fig. S1. Analytical procedure for the determination of Pu in soil samples.
